# The Making of Leaves: How Small RNA Networks Modulate Leaf Development

**DOI:** 10.3389/fpls.2018.00824

**Published:** 2018-06-18

**Authors:** Tianxiao Yang, Yongyan Wang, Sachin Teotia, Zhanhui Zhang, Guiliang Tang

**Affiliations:** ^1^National Key Laboratory of Wheat and Maize Crop Science, Collaborative Innovation Center of Henan Grain Crops, College of Agronomy, Henan Agricultural University, Zhengzhou, China; ^2^Department of Biological Sciences, Michigan Technological University, Houghton, MI, United States; ^3^Department of Biotechnology, Sharda University,Greater Noida, India

**Keywords:** *Arabidopsis thaliana*, leaf development, small RNAs, target genes, regulatory network

## Abstract

Leaf development is a sequential process that involves initiation, determination, transition, expansion and maturation. Many coding genes and a few non-coding small RNAs (sRNAs) have been identified as being involved in leaf development. sRNAs and their interactions not only determine gene expression and regulation, but also play critical roles in leaf development through their coordination with other genetic networks and physiological pathways. In this review, we first introduce the biogenesis pathways of sRNAs, mainly microRNAs (miRNAs) and trans-acting small interfering RNAs (ta-siRNAs), and then describe the function of miRNA-transcription factors in leaf development, focusing on guidance by interactive sRNA regulatory networks.

## Introduction

Leaf is one of the most important organs in flowering plants because of its essential roles in photosynthesis (Efroni et al., 2010). The leaf lamina is typically broad, flat, and thin, and differentiates into the upper (adaxial) surface and the lower (abaxial) surface (Braybrook and Kuhlemeier, 2010). In most plant species, the upper surface facilitates light capture, whereas the lower surface facilitates gas exchange and regulation of transpiration (Husbands et al., 2009; Yamaguchi et al., 2012). Leaf morphology varies greatly among species and within different developmental stages and growth conditions (Rodriguez et al., 2014). Leaf shapes and sizes not only determine plant architecture, but also fundamental agronomic traits for crop productivity.

Small RNAs (sRNAs), including microRNAs (miRNAs) and small interfering RNAs (siRNAs), are 18–30 nt long, endogenous, non-coding RNAs. While substantial differences in the size of sRNAs have been documented, miRNAs and siRNAs have their own typical sizes. sRNAs play important roles in development, metabolism and chromatin modifications (Chen, 2009). In plants, the biogenesis of miRNAs comprises of several sequential steps, including *MIRNA* transcription, nuclear processing, nuclear export, cytoplasmic processing, and RNA-induced silencing complex (RISC) assembly. The ARGONAUTE 1 (AGO1) protein recruits mature miRNAs in the assembly of RISCs, thereby to cleave target mRNA or block its translation (Rogers and Chen, 2013; Iwakawa and Tomari, 2015).

Trans-acting siRNA (ta-siRNA) is a miRNA-derived siRNA. In *Arabidopsis*, miR173 initiates the production of TAS1a-c and TAS2 siRNA, miR390 of TAS3, and miR828 of TAS4 siRNA (Allen et al., 2005; Allen and Howell, 2010). The production of ta-siRNA involves two distinct pathways: the ‘one-hit’ and the ‘two-hit’ system (Vazquez et al., 2004; Axtell et al., 2006). The ‘one-hit’ system requires one 22-nt miRNA (miR173 and miR828) and one AGO1 protein, whereas the ‘two-hit’ system requires two 21-nt miRNAs (miR390) and two AGO7 proteins (Chapman and Carrington, 2007).

Compared to animal miRNAs, plant miRNAs have fewer targets. Plant target genes usually encode transcription factors (TFs) and F-box proteins, which places miRNAs at the center of plant gene regulatory networks (Rubio-Somoza and Weigel, 2011). A number of sRNAs and their target TFs have been shown to determine leaf development, such as miR156-*SQUAMOSA PROMOTER BINDING PROTEIN-LIKE* (*SPL)* (Xu et al., 2016), miR160-*AUXIN RESPONSE FACTOR* (*ARF)* (Ben-Gera et al., 2016), miR165/166-Class III *HOMEODOMAIN-LEUCINE ZIPPER* (*HD-ZIPIII*) (Jia et al., 2015), miR319-*TEOSINTE BRANCHED/CYCLOIDEA/PROLIFERATING CELL FACTOR* (*TCP)* (Bresso et al., 2018), miR390-*TRANS-ACTING SMALL INTERFERING RNA3* (*TAS3*) (Husbands et al., 2015) and miR396-*GROWTH REGULATING FACTOR* (*GRF*) (Omidbakhshfard et al., 2015). Additionally, multiple crucial components that are required for miRNA and ta-siRNA biogenesis, such as *AGO1* (Bohmert et al., 1998), *AGO7* (Garcia et al., 2006), *SERRATE* (*SE*) (Prigge and Wagner, 2001), and *HYPONASTIC LEAVES 1* (*HYL1*) (Liu et al., 2011), have been suggested to influence leaf development.

**FIGURE 1:**
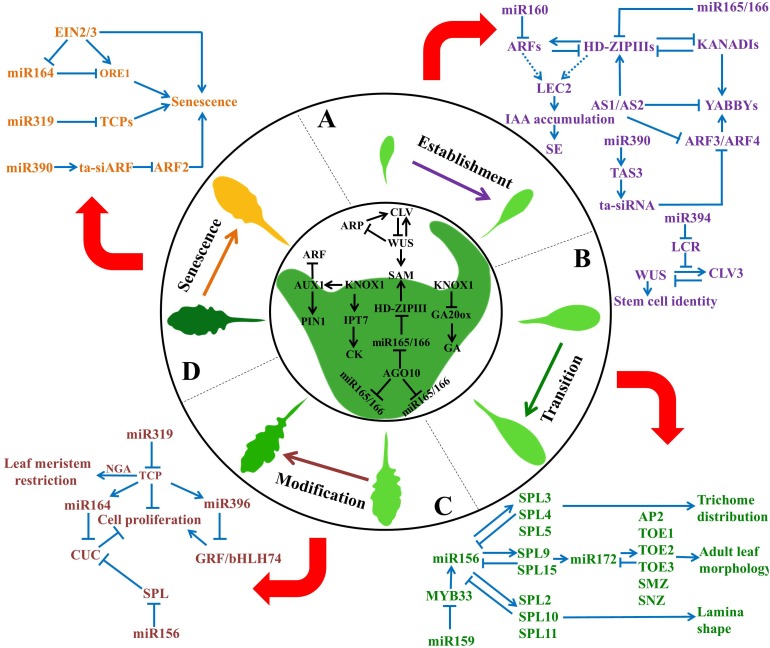
Overview of miRNA-mediated regulatory networks in control of leaf development. Leaf development is a complex biological process that is tightly controlled by miRNAs and their target genes. It consists of four stages: **(A)** estabishment, **(B)** transition, **(C)** modification, and **(D)** senescence. sRNA networks play an important role in each stage of leaf development. For instance, miR160 and miR165/166 regulate leaf initiation; miR165/166 and miR390 control leaf polarity; miR164 and miR319 modulate leaf morphology; miR156 and miR172 determine phase transition; miR164 and miR319 contribute to leaf senescence. Blue arrows indicate positive regulation, while T shaped bars indicate negative regulation. SE, somatic embryogenesis.

Leaf development is a complex process that involves the connections and interactions of many miRNA-pathway components to generate multilayered networks. In this review, we discuss current knowledge of the sRNA-mediated regulatory networks that function during leaf developmental processes, including leaf initiation, polarity determination, phase transition, leaf morphology and leaf senescence (**Figure 1**). First, we briefly introduce the formation of mature leaves and provide an overview of stages of leaf morphogenesis. Next, we describe the molecular controls of leaf development. In particular, we focus upon miRNA-TF and miRNA-miRNA interactions involved in this multifaceted sequence of leaf development. Finally, we highlight several important aspects that should be addressed to fully understand the functional complexity of such miRNA networks.

## The Developmental Processes of Leaf Morphogenesis

Leaves arise from the flanks of the shoot apical meristem (SAM), establish proximo-distal, adaxial-abaxial and medio-lateral axes, followed by subsequent cell division and expansion to reach their final size and shape. Three regulatory pathways which are involved in the establishment of leaf polarity and SAM maintenance are: *CLAVATA* (*CLV)*-*WUSCHEL* (*WUS)*, *KNOTTED-LIKE HOMEOBOX* (*KNOX*)-*ASYMMETRIC LEAVES1* (*AS1*) in *Arabidopsis*, *ROUGH SHEATH2* (*RS2*) in maize and *PHANTASTICA* (*PHAN*) in snapdragon, together known as the *AS1/RS2/PHAN* (*ARP)* pathway (Waites et al., 1998; Timmermans et al., 1999; Guo et al., 2008; Tabata et al., 2010). Furthermore, HD-ZIP III-KANADI proteins sequentially operate upon three axes to ensure the robust growth and development of leaves. The generation of leaves is largely governed by the repression of Class I *KNOX* (*KNOXI)* genes via two pathways. One is the plant hormone dependent pathway, in which auxin polar transport and local accumulation determines the site of leaf primordium initiation, while a low cytokinin (CK)/gibberellin (GA) ratio promotes SAM maintenance (Reinhardt et al., 2000; Fleishon et al., 2011). The other is the *ARP* genes*-*dependent pathway. *ARP* genes encode MYELOBLASTOSIS (MYB)-domain TFs and are expressed in lateral organ founder cells. AS1 recruits a repressor complex with the LATERAL ORGAN BOUNDARIES domain protein, AS2, which binds to the promoter region of *KNOX* genes (Guo et al., 2008). Thus, leaf development is controlled temporally and spatially by several pivotal factors, such as plant hormones, TFs and sRNAs (Pulido and Laufs, 2010; Byrne, 2012; Dkhar and Pareek, 2014; Kalve et al., 2014).

## The Interactions of miRNAs Involved in Leaf Development

### The Interplay of miR160, miR165/166, and miR394 Regulates Leaf Initiation in Plants

sRNA transcriptome profiling data revealed that early embryonic development involves the function of several miRNAs, such as miR156, miR160, miR166, miR167, miR390, and miR394 (**Figure 1A**) (Armenta-Medina et al., 2017). Two miRNA interaction nodes greatly affect meristem maintenance and leaf initiation: the module of miR394 and *LEAF CURLING RESPONSIVENESS* (*LCR*) mediated non-cell autonomous pathway, and the interplay of the miR160 and miR165/166-mediated cell autonomous pathway. The protoderm-specific miR394 represses the *LCR* gene expression to confer stem cell maintenance, which also involves a local feedback loop mediated by *WUS* and *CLV* (Knauer et al., 2013).

*ARF10*, *ARF16*, and *ARF17*, which are the targets of miR160, are required for the maintenance of local auxin peaks in the meristem, and phyllotaxis in the rosette (Pulido and Laufs, 2010). *Arabidopsis* transgenic plants expressing a miR160-resistant version of *ARF10* or *ARF17* exhibited pleiotropic phenotypes, including abnormal numbers and positions of cotyledons and up-curled, strongly serrated rosettes, indicating that miR160 and ARFs account for leaf initiation (Mallory et al., 2005; Liu et al., 2007). Additionally, miR165/166 is suggested to regulate the organization and maintenance of the SAM. AGO10 sequestrates miR165/166 and thus protects HD-ZIP III transcripts from degradation. AGO10 competes with AGO1 for loading miR165/166 into AGO1-guided RISC complex. This de-represses the expression of HD-ZIP III family genes which helps in SAM maintenance (Zhu et al., 2011). *AGO10* gain-of-function mutants displayed varying degrees of leaf hyponasty and serration, whereas loss-of-function of *AGO10* resulted in the formation of pin-headed or even empty apexes (Roodbarkelari et al., 2015; Yu et al., 2017). The interaction between miR160 and miR165/166 contributes to somatic embryogenesis via activation of *LEAFY COTYLEDON2* (*LEC2*)-mediated auxin biogenesis pathways. Their targets, *ARFs* and *HD-ZIP IIIs*, also interact with each other via a negative feedback loop to further modulate *LEC2* expression. HD-ZIP III proteins, PHAVOLUTA (PHV), and ARF10/ARF16 activate *LEC2* expression, thereby inducing the up-regulation of several key genes of the auxin synthesis pathway which ultimately triggers indole-3-acetic acid (IAA) accumulation (Wójcik et al., 2017). These findings provide a detailed insight into how miR160-*ARF*, miR165/166-*HD-ZIPIII* and miR394-*LCR* nodes control somatic embryogenesis, meristem maintenance and leaf initiation.

### The Interplay of miR165/166, miR390, and miR396 Controls Leaf Polarity in Plants

The establishment of leaf polarity is an important event in leaf development, and several TFs and sRNAs have been proposed to be involved in pattern identity via antagonistic interactions (**Figure 1A**). Generally, opposite surfaces of the leaf are maintained by two polarity regulators: the adaxial determiners and the abaxial determiners. *HD-ZIPIIIs* and *AS1/AS2* are adaxial determiners where *AS1/AS2* promote *HD-ZIPIIIs*. *KANADIs* and *YABBYs* are abaxial determiners, where *KANADIs* activate *YABBYs* (Chitwood et al., 2007; Yamaguchi et al., 2012). Additionally, *HD-ZIP IIs* are involved in the regulation of polarity identity by repressing miR165/166 (Merelo et al., 2016).

Accumulating data have suggested that miR165/166 and miR390-derived ta-siRNAs determine leaf polarity. miR165/166 targets encode five of the HD-ZIP III TFs, including PHB, PHV, REVOLUTA (REV), ATHB-8 and ATHB-15. Of these, *PHB, PHV*, and *REV* act redundantly to promote adaxial identity. miR390, which is derived from four loci (*MIR390 a-d*) of the *Arabidopsis* genome, also contributes to leaf polarity through the ta-siRNA-dependent pathway. miR390-directed *TAS3* cleavage leads to the production of ta-siRNAs which further target *ARF3* and *ARF4* transcripts to regulate leaf abaxial identity (Pekker et al., 2005). Furthermore, *AS1*/*AS2* repress *ARF3*/*ARF4* to stabilize adaxial-abaxial partitioning in three ways by: directly binding to the promoter region of *ARF3*, indirectly activating miR390- and RDR6-dependent pathway leading to silencing of *ARF*3/*4*, and maintaining DNA methylation in the coding regions of *ARF3* (Iwasaki et al., 2013).

Both miR165/166 and miR390 have been shown to move between cells and to interact with each other during the formation of leaf developmental boundaries (Marin-Gonzalez and Suarez-Lopez, 2012). *HD-ZIP IIIs* and *ARF3/4* are required for the fate of the leaf upper and lower surface, respectively. miR165/166 moves from the bottom to top to establish a gradient along the axis, which defines a stable boundary, and limits the localization of *HD-ZIP III* expression. A similar but opposite pattern was also observed for *TAS3-ARF*. The movement of miR165/166 and miR390 generates sharp boundaries via a threshold-based readout of their mobility gradients (Robinson and Roeder, 2017; Skopelitis et al., 2017).

In addition to the well-known modules of miR165/166 and miR390 controlling leaf polarity, an interesting study by Das Gupta and Nath (2015) revealed that divergent leaf growth polarity is strongly correlated to the miR396-*GRF* expression gradient. Positive allometry, or basipetal growth occurs when the growth rate of the base is faster than that at the tip and vice versa for negative allometry, or acropetal growth. The accumulation of miR396 is higher near the tip but lower near the base in basipetal leaves, whereas acropetal leaves exhibit an opposite expression pattern. Consistent with this, the expression level of miR396 is uniform throughout the leaves with diffused growth (Das Gupta and Nath, 2015). Taken together, leaf polarity is regulated in a quantitative manner that involves sRNA production, movement and action.

### The Interplay of miR156, miR159, and miR172 Determines Phase Transition in Plants

Plants undergo several developmental changes during their life cycles, including two phase changes, from juvenile to adult and from vegetative to reproductive (Poethig, 2009). Leaf morphology also changes during these transitions, and is characterized by trichome initiation, severe serration, and blade alteration (Usami et al., 2009). Two conserved miRNAs, miR156 and miR172, are involved in phase transition (**Figure 1B**) (Wu et al., 2009). In *Arabidopsis*, the targets of miR156/157 encode 10 SPL TFs, SPL2-6, SPL9-11, SPL13, and SPL15. However, the targets of miR172 encode six of the APETALA2 (AP2)-like TFs, including TARGET OF EAT1 (TOE1), TOE2, TOE3, SCHLAFMUTZE (SMZ), and SCHNARCHZAPFEN (SNZ). The targets of miR156 involve several pathways that act redundantly to control leaf phase change. First, *SPL3*/*4/5* determine trichome formation and distribution; second, *SPL9*/*15* modulate adult leaf morphology, of when miR156 interacts with miR172 via *SPL9* and *SPL10*. Finally, *SPL2/10/11* regulate the lamina shape and act independently of miR172 (Huijser and Schmid, 2011).

A regulatory model has demonstrated the interaction between miR156 and miR172 (Teotia and Tang, 2015). miR156 is controlled by the positive feedback loop of *SPL9*, whereas the negative feedback loop is modulated by *SPL15*. Additionally, miR156 is positively regulated by AP2 and AGAMOUS-LIKE15 (Wu et al., 2009; Serivichyaswat et al., 2015). Furthermore, expression of miR172 is fine-tuned by the positive feedback loop of *TOE1/2* and negatively by *AP2* via *LEUNIG* (*LUG*) and *SEUSS* (*SEU*) (Yant et al., 2010; Grigorova et al., 2011). TOE1/2 repress *SPL3/4/5*, while SPL3 activates *TOE3* expression. In addition to the regulatory nodes of miR156 and miR172, miR159 acts as a modifier to control the timing of juvenile to adult transition. *MYB33*, a target of miR159, directly binds to the promoters of *MIR156A*, *MIR156C*, and *SPL9* and activates their expression (Guo et al., 2017).

### The Interplay of miR164, miR319, and miR396 Modulates Leaf Morphology in Plants

Leaf shape varies greatly among different plant species which is largely attributed to the level and pattern of dissection in the primordium margin. Recent studies have shown miR164 and miR319 as key regulators in leaf shaping (**Figure 1C**). PIN-FORMED 1 (PIF1) and CUP-SHAPED COTYLEDON (CUC) TFs modify marginal serrations in leaf. The inactivation of *CUC2* resulted in reduced serrations and smooth margins, while *CUC2* overexpression led to the formation of highly serrated leaves. *CUC2* is a target of miR164 and its expression level determines the level of serration in the *Arabidopsis* leaf (Nikovics et al., 2006). Moreover, CUC1 and CUC3 act redundantly in the formation of incipient serrations in leaves. CUC2 interacts with CUC3 in a feed-forward mechanism. CUC3 gets activated by the CUC2 homodimer, then CUC2 and CUC3 heterodimerize to promote leaf complexity (Rubio-Somoza et al., 2014).

miR319, also known as miRJAW in *Arabidopsis*, regulates leaf complexity by targeting multiple *TCP* genes (Palatnik et al., 2003). The overexpression of miR319 promoted the formation of additional serrations whereas MIM319 plants, that repress endogenous miR319 activity, showed opposite phenotypes (Rubio-Somoza et al., 2014). The study of a series of *MIR319* and *TCP* mutants revealed their important roles in cotyledon boundary establishment, leaf serration, and the onset of leaf senescence (Koyama et al., 2017). Furthermore, multiple TCP TFs coupled with NGATHA (NGA) TFs are shown to be involved in sequential developmental processes, including meristem activity localization, marginal persistent differentiation and adaxial-abaxial maintenance (Alvarez et al., 2016).

miR164-*CUC* and miR319-*TCP* modules function in a coordinated manner for leaf size regulation. *TCP3*, a miR319 target, was found to negatively regulate *CUC2* by directly activating *MIR164A* (Koyama et al., 2007). TCPs can also interfere with CUC2 in a miR164-independent manner, resulting in a serration-deficient phenotype. Furthermore, *SPLs*, targeted by miR156, not only act as timing cues but also regulate *TCP-CUC* interactions. For example, SPL9 dimerizes with TCP4 and competes with CUC2/3 for TCP4 interaction. This links the temporal development regulator (*SPL*) with factors responsible for leaf margin modification (*CUC*) via a common player of leaf cell proliferation and differentiation (*TCP*) (Chitwood and Sinha, 2014; Rubio-Somoza et al., 2014).

Leaf shape is also modulated by *GRFs*, which are involved in cell division and expansion. *GRF1/2/3* loss-of-function mutants exhibited smaller leaf size, while plants overexpressing them produced bigger leaves (Kim et al., 2003). During leaf development, expression of miR396 increases and that of target *GRF* decreases, which acts as a signal to determine final leaf size (Rodriguez et al., 2010). GRF-interacting factor 1 (GIF1), interacts with both chromatin-remodeling complexes and GRF proteins, thereby maintaining the cell proliferation activity of vegetative and reproductive organ primordium (Debernardi et al., 2014; Kim and Tsukaya, 2015). An additional target for miR396b, basic helix-loop-helix DNA-binding superfamily protein 74 (bHLH74), also contributes to leaf margin and vein pattern formation (Debernardi et al., 2012). Intriguingly, Mecchia et al. (2013) proposed that the miR396 regulatory pathway cooperates with the ta-siRNA biogenesis pathway in leaf morphogenesis. *TCPs* simultaneously promote miR164 and miR396, which have opposing functions in leaf cell proliferation (Schommer et al., 2014). Thus, *GRFs*, negatively regulated by miR396, also lie within this regulatory network, which is guided by miR156 and centered in miR319.

### The Interplay of miR164, miR319, and miR390 Contributes to Leaf Senescence in Plants

Leaf senescence is the final stage of leaf development which involves distinctive actions at the cellular, tissue, organ, and organism levels (Lim et al., 2007). miR164 and miR319 are involved in controlling leaf senescence (**Figure 1D**). A target of miR164, *ORESARA1* (*ORE1*), functions as a positive regulator of aging-induced cell death and leaf senescence in *Arabidopsis*. *ORE1* is induced in an age-dependent manner by *ETHYLENE INSENSITIVE2* and *3* (*EIN2/3*), central components of ethylene signaling (Li et al., 2013). *ORE1* expression is gradually down-regulated by miR164 during earlier stages of leaf development, but is up-regulated at later stages by *EIN2/3*. Additionally, *EIN2/3* mediate aging-induced cell death in the absence of both miR164 and *ORE1*. The trifurcate feed-forward pathway provides a highly robust regulation to ensure coupled leaf senescence and cell death (Kim et al., 2009).

miR319, which targets the *TCP* genes, is also proposed to be a key player in leaf senescence. *TCP4* has been shown to activate the jasmonic acid (JA) biosynthetic gene *LIPOXYGENASE2*, which increases levels of JA, and accelerates leaf senescence (Schommer et al., 2008). These findings showed that miR319-*TCPs* control two different processes of leaf development by negatively regulating growth but positively regulating senescence (Schommer et al., 2008). In addition to ethylene and JA, auxin also plays an important role in the control of plant longevity. *ARF2*, a negative regulator of auxin responses, is believed to regulate leaf senescence (Ellis et al., 2005; Lim et al., 2010). These findings suggest the importance of exploring the complex regulatory networks comprising of miR164/*ORE1*, miR319/*TCP*, and miR390/*TAS3*/*ARF2* modules.

## Conclusion and Perspectives

In recent years, considerable advances have been made in our understanding of sRNA-mediated regulatory networks in leaf development. In this review, we have provided an overview on miRNA interactions during leaf development. We have emphasized on how sRNAs participate in regulatory networks, involved in various stages of leaf development. We showed that sRNAs can have very specific roles (for example miR160 in leaf initiation) and also very broad roles (for example miR319 in leaf expansion and senescence). Additionally, we explored how different targets of the same miRNA regulate diverse processes (for example miR164 targets, *CUC2* in leaf morphology and *ORE1* in leaf senescence).

The discovery of more miRNA interacting networks for leaf development, will aid in deciphering important leaf-related traits through manipulation of miRNA/target gene expression. However, our knowledge of sRNA-mediated leaf development networks is far from complete. The systematic study of their roles should integrate several strategies to reconstitute the developmental pathway of single cells from the leaf primordium to their final position in the mature leaf. Such explorations will benefit from the increasing next-generation sequencing techniques, bioinformatics analysis tools, and maturing mathematical modeling methods. Further research could help apply the knowledge gained from the model organisms to crop plants for modulation of leaf shape to promote photosynthesis and increase biomass and yield.

## Author Contributions

GT and ZZ conceived the topic and revised the manuscript. TY and YW wrote the first draft of the manuscript and drew the figure. ST further modified the manuscript and the figure with intellectual inputs. All authors read and approved the final manuscript.

## Conflict of Interest Statement

The authors declare that the research was conducted in the absence of any commercial or financial relationships that could be construed as a potential conflict of interest.
